# Supplementary material for: Seroepidemiological Survey of Hepatitis E Virus in Intensive Pig Farming in Vojvodina Province, Serbia
Source: Animals (Basel). 2025 Jan 9;15(2):151. doi: 10.3390/ani15020151 (PMC11758650; doi:10.3390/ani15020151)
Supplement: Supplementary file 1 [file animals-15-00151-s001.zip › animals-3387267-supplementary.pdf]

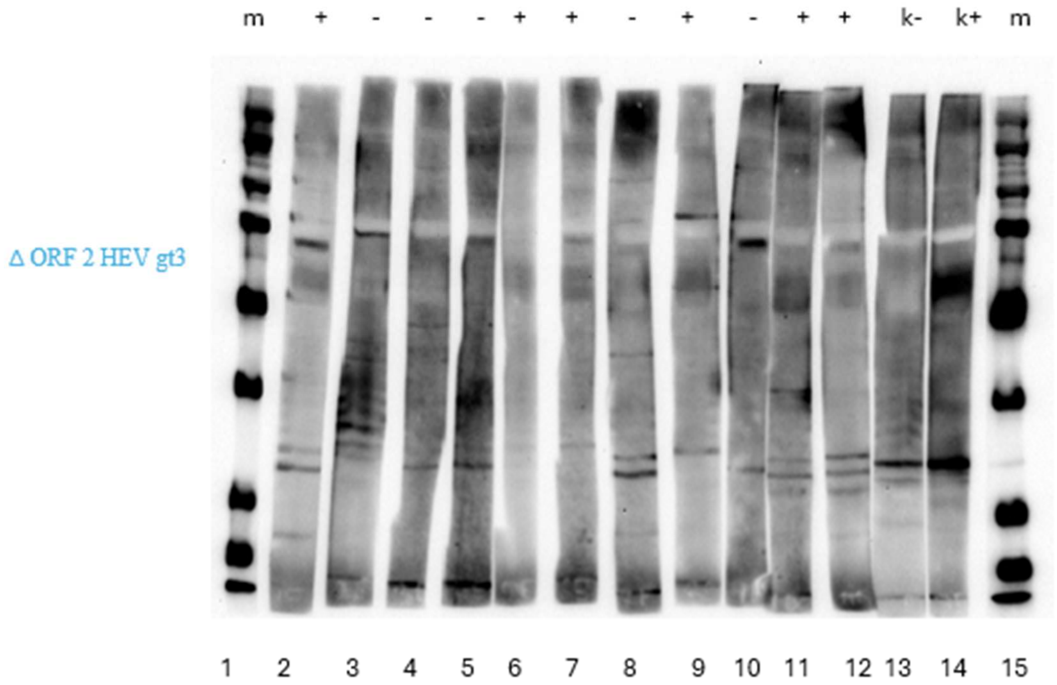

Figure S1: Results of Western blot for pig sera: marker is placed in the first and last line (1st and 15th column); from 2 to 12 columns are added eleven examined swine sera; negative and positive HEV controls are placed on the position 13 and 14, respectively. Above the test sera are the marks "+" or "-" which means that the result is positive or negative in the Western blot technique (appearance of a molecular mass band of 65-68 kDa). Based on the obtained results, positive or negative test results for the presence of antibodies against HEV with in-house ELISA were defined.
